# Commentary on: Screening of immunosuppressive cells from colorectal adenocarcinoma and identification of prognostic markers

**DOI:** 10.1042/BSR20211096

**Published:** 2021-12-17

**Authors:** Kexin Chen, Zhen Zeng, Chunxiang Ma, Yuan Dang, Hu Zhang

**Affiliations:** 1 Department of Gastroenterology, West China Hospital, Sichuan University, Chengdu, China; 2 Centre for Inflammatory Bowel Disease, West China Hospital, Sichuan University, Chengdu, China

**Keywords:** biomarker, colorectal adenocarcinoma, Gene Expression Omnibus, Th17 cells, The Cancer Genome Atlas

## Abstract

Colorectal adenocarcinoma (COAD) is one subtype of colorectal carcinoma (CRC), whose development is associated with genetics, inappropriate immune response, and environmental factors. Although significant advances have been made in the treatment of COAD, the mortality rate remains high. It is a pressing need to explore novel therapeutic targets of COAD. Available evidence indicated that immune cell infiltration was correlated with cancer prognosis. To reveal the roles of immune cells in the COAD prognosis, a study published in Bioscience Reports by Li et al. (Bioscience Reports (2021) **41**, https://doi.org/10.1042/BSR20203496) analyzed data from The Cancer Genome Atlas (TCGA) and Gene Expression Omnibus (GEO) dataset. It demonstrated a beneficial effect of Th17 cells in COAD prognosis. In addition, six hub genes (*KRT23, ULBP2, ASRGL1, SERPINA1, SCIN*, and *SLC28A2*) were identified to correlate with Th17 cells and COAD prognosis, suggesting one new therapy strategy and some predictive biomarkers of COAD. These findings reported by Li et al. may pave one way to explore the molecular mechanism of COAD further.

First, the present study was based on Cancer Genome Atlas (TCGA) and Gene Expression Omnibus (GEO). TCGA was established in 2006, contains various kinds of cancer data, including clinical data, cancer genome data, mRNA expression data, and epigenetic data. Based on TCGA data, some significant findings have been made for colorectal adenocarcinoma (COAD), including similar genomic profiles in the colon and rectal cancers, hypermutated subtype associated with favorable prognosis. In addition, some hub genes also were identified, like *ARID1A, SOX9, FAM123B/WTX* [1], *TM4SF1* [2] in TCGA. These hub genes to be further studied and get some significant achievements: (i) the low expression of the *ARID1A* in COAD tissue indicated poor COAD prognosis [3]; (ii) the up-regulation of the *SOX9* facilitated the growth of colorectal carcinoma (CRC) cells for promoting CRC progression [4]; (iii) *WTX* loss is tightly associated with the CRC proliferation and liver metastasis [5]; and (iv) the high expression of *TM4SF1* in CRC tissue enhances tumor proliferation, invasion, and migration [2]. Such evidence desires to provide an inspiring therapeutic target and screen a reliable prognostic biomarker for CRC. However, the hub genes that act in the pathological process of CRC are not fully elucidated, the function and substantial mechanism of these genes also need further studies, and there is still a lengthy procedure before they are used to forecast the clinical prognosis. Thus, the study presented by Li et al. [11] is worthwhile to explore the genetic mutation profile of CRC and give implications for further studies in CRC regarding therapy and prognosis.

Second, according to the recent studies regarding tumor-infiltrating immunosuppressive cells in CRC, these cells can be departed into myeloid-derived cells (dendritic cell, macrophage, myeloid-derived suppressor cell) and lymphoid-derived cells (Treg cell, B cell, NK cell), in which two sorts of cells interact with the suppressive function of each other [6,7]. As an example, myeloid-derived suppressor cells (MDSCs) restrict the proliferation of T cells and promote the development of the Treg to support tumor development [8]. Of note, Th17 cell, which is one of the T-cell subsets, had been extensively researched, but the distinct effect of these cells in CRC prognosis is controversial. Some evidence indicated that CRC patients with high expression of Th17 cluster had a poor prognosis [9]. Conversely, the protective role of Th17 cells in CRC was reported [10]. Li et al. [11] showed that the Th17 cells imbue a protective effect on COAD. This finding can better understand the Th17 cells involved in COAD and trigger a deep thought in related research. Intriguingly, opposite effects of Th17 cells in CRC prognosis may contribute to the diverse location and cytokines of the Th17 cells [12,13]. Further studies are required to uncover the function of Th17 in a more detailed way.

Third, in the study of Li et al. [11], immune cells infiltrated in COAD samples are proposed to be responsible for the prognosis of patients. By using Cox univariate analysis, the function of Th17 cells in COAD prognosis had been demonstrated. Notably, six hub genes (*KRT23, ULBP2, AS-RGL1, SERPINA1, SCIN*, and *SLC28A2*) were found first to progress Th17 cells regulating COAD prognosis. A particularly novel aspect of the present study is that the hub genes were associated with the Th17 cells. Their function in COAD diagnosis and prognosis was proved. Consistent with the findings of Li et al., previous researches suggested that *ULBP2, SCIN*, and *KRT23* could be used as a diagnostic and prognostic biomarker of COAD in light of its high expression in the cancer tissues [14–16]. Besides, elevated level of *SERPINA1* expression in serum samples of CRC patients was associated with disease progression [15]. Thus, these studies indicated the potential of six hub genes found by Li et al. as a biomarker for CRC prognosis, and further research needs to explore these revealed hub genes regarding Th17 cells and their effects on COAD prognosis [11].

Fourth, accumulating evidence has shown that epigenetic modifications, including DNA methylation, histone modifications, and non-coding RNAs, significantly contributed to cancer diagnosis, treatment, and prognosis. Jung et al. [17] also claimed that some epigenetic alterations (for example, methylation of the *SEPT9*, *SHOX2*, *ALX4*, and *VIM*) were reliable biomarkers for diagnosing CRC. More importantly, the methylation of the *SEPT9* is approved by FDA as a biomarker for CRC screening [18]. However, no epigenetic biomarker has been used in clinical practice to predict the prognosis or the immunotherapy effects of CRC. More studies are needed to fill this gap. Given that these findings were made based on small sample databases, it is certainly warranted to verify its biological functions and associated pathways in cell and animal studies. Therefore, function enrichment analysis has also become highly significant.

Finally, a growing number of publications have focused on the function of the immunosuppressive cells in the immunotherapy of CRC. Treg cells infiltrating in tumor tissue enhance the chemoresistance to 5-FU to influence the prognosis of patients [19]. Similarly, Th17 cells can inhibit CD8^+^T cells infiltration, thus reducing the efficiency of immunotherapy [20]. However, the study regarding the role of T cells in immunotherapy is not to be fully explored. Of note, immunotherapy resistance-associated genes were found. One research reported that the *KRAS* gene mutations were associated with a low effective rate of targeting epidermal growth factor receptor (EGFR) immunotherapy [21]. Besides, acquired *B2M* gene mutations in CRC led to a resistance to programmed cell death (PD-1)-blocking antibodies (pembrolizumab) [22,23]. Likewise, Wang et al. summarized the mutation genes responsible for the efficacy of the immunotherapy, including *KRAS*, *TP53*, *NRAS, PBRM1*, and *JAK* [24]. So further studies need to address the relationship of those genes with immunosuppressive cells. Li et al. revealed Th17 cell-associated genes in COAD prognosis, which provide an inspiring target to improve the efficacy of immunotherapy. This underscores the importance of further studies to delineate precise and novel immunotherapy of COAD better and help explain the underlying COAD’s molecular mechanism modifications, guiding the clinical doctors to treat the immunotherapy that targets the EGFP and PD-1 carefully.

## Abbreviations

COAD: colorectal adenocarcinoma
CRC: colorectal carcinoma
GEO: Gene Expression Omnibus
PD-1: programmed cell death 1
TCGA: The Cancer Genome Atlas
